# Anodal Transcranial Direct Current Stimulation Over Prefrontal Cortex Slows Sequence Learning in Older Adults

**DOI:** 10.3389/fnhum.2022.814204

**Published:** 2022-02-24

**Authors:** Brian Greeley, Jonathan S. Barnhoorn, Willem B. Verwey, Rachael D. Seidler

**Affiliations:** ^1^Department of Physical Therapy, University of British Columbia, Vancouver, BC, Canada; ^2^Department of Learning, Data-Analytics and Technology, University of Twente, Enschede, Netherlands; ^3^Department of Applied Physiology and Kinesiology, University of Florida, Gainesville, FL, United States

**Keywords:** tDCS, older adults, prefrontal cortex, motor sequence learning, reaction time, chunking, explicit learning, learning impairment

## Abstract

Aging is associated with declines in sensorimotor function. Several studies have demonstrated that transcranial direct current stimulation (tDCS), a form of non-invasive brain stimulation, can be combined with training to mitigate age-related cognitive and motor declines. However, in some cases, the application of tDCS disrupts performance and learning. Here, we applied anodal tDCS either over the left prefrontal cortex (PFC), right PFC, supplementary motor complex (SMC), the left M1, or in a sham condition while older adults (*n* = 63) practiced a Discrete Sequence Production (DSP), an explicit motor sequence, task across 3 days. We hypothesized that stimulation to either the right or left PFC would enhance motor learning for older adults, based on the extensive literature showing increased prefrontal cortical activity during motor task performance in older adults. Contrary to our predictions, stimulation to the right and left PFC resulted in slowed motor learning, as evidenced by a slower reduction rate of reduction of reaction time and the number of sequence chunks across trials relative to sham in session one and session two, respectively. These findings suggest an integral role of the right PFC early in sequence learning and a role of the left PFC in chunking in older adults, and contribute to mounting evidence of the difficultly of using tDCS in an aging population.

## Introduction

Aging is associated with declines in sensorimotor function (Raz, 2000; Seidler, 2006). Given that sensorimotor function plays a role in activities of daily living, declines can translate into a loss of independence. Physical rehabilitation approaches to mitigate these declines largely rely upon motor learning based interventions. While these interventions result in some improvements, the addition of non-invasive brain stimulation has been shown to make them more effective (Patel et al., 2019). Transcranial direct current stimulation (tDCS) has emerged to be especially promising because of its low cost, safety, portability, and its ability to be successfully used at home with clinical (Charvet et al., 2015; Kasschau et al., 2016) and older adult (Ahn et al., 2019) populations.

Older adults typically learn new motor skills at a slower rate than young adults. Verwey (2010) found that when practicing a sequence of finger presses to a repeated sequence of targets, older adults exhibited poorer performance for the repeated sequence, as indicated by slower reaction time. Similarly, Brown et al. (2009) found that while older adults displayed motor sequence learning during a single session (online gains), when participants were re-tested 24 h later, young adults exhibited a beneficial, between-session consolidation effect (offline gains) while older adults did not. These findings are consistent with the idea that neural plasticity and consolidation may be reduced with advancing age (Wilhelm et al., 2008, 2012). Thus, previous research shows that despite age-related declines in the sensorimotor system, older adults can learn new motor tasks, albeit not as well as young adults.

The use of tDCS paired with motor practice in older adult populations shows promise for ameliorating age-related motor declines. In a five session study during which older adults practiced a serial reaction time task paired with anodal (typically excitatory) tDCS, researchers found that those who received M1 anodal tDCS exhibited greater sequence specific learning relative to those who received sham stimulation (Dumel et al., 2016). In another study, young and older adult participants received stimulation over M1 while learning a sequence of finger movements (Zimerman et al., 2013). Without stimulation, older adults demonstrated poorer motor performance relative to young adults. However, the older adult participants who received stimulation during practice no longer had a motor performance deficit. While the findings from these studies suggest that tDCS may improve motor learning for older adults, other studies show either no benefit or even poorer performance when tDCS is paired with task practice (Mooney et al., 2019; Muffel et al., 2019; Habich et al., 2020; Chow et al., 2021). For example, one study found no benefit of a single session of anodal M1 tDCS as older and young adult participants learned a sequential isometric force task using their non-dominant hand during practice (Mooney et al., 2019).

For tDCS to be considered a viable therapeutic option for older adults, the optimization of tDCS parameters such as intensity, timing, number of sessions, etc., is necessary. For example, a recent study used a computational model to show that older adults required a slightly higher stimulation intensity (i.e., ∼2.3 mA) to achieve the same current density as in young adults with 2 mA of tDCS over the left primary motor cortex (Indahlastari et al., 2020). Alternatively, the timing of the application of tDCS relative to task practice, the task specificity of tDCS, or a combination may explain the inconsistencies frequently observed in older adult populations. In a cognitive naming task, older adults that received anodal tDCS over the left prefrontal cortex (PFC) during task execution displayed significant improvements relative to sham, whereas older adults that received tDCS before the task showed no differences compared to sham; in contrast, young adults that received stimulation before or during task practice showed task improvements (Fertonani et al., 2014). In another study, older adults that received anodal tDCS over M1 immediately following training of a motor sequence task, but not an hour or 2 h after, showed enhanced consolidation (Rumpf et al., 2017). The number of active tDCS sessions also likely impacts the effectiveness of tDCS, with multi-session protocols appearing to be more effective than a single session of tDCS (Hashemirad et al., 2016; Talsma et al., 2017; Shekhawat and Vanneste, 2018; Song et al., 2019). Overall, these studies suggest that tDCS in older adult populations has potential for reducing motor and cognitive declines, but the benefits may be task dependent (Saucedo Marquez et al., 2013; Kimura et al., 2021), and require tDCS parameter optimization such as intensity (Indahlastari et al., 2020) or timing (Fertonani et al., 2014; Rumpf et al., 2017) relative to task practice. However, little is known about how anodal tDCS over other brain regions outside of M1 affects motor learning in older adults.

Non-invasive brain stimulation studies have uncovered a complex role of the prefrontal cortices (PFC) in motor sequence learning. Janacsek et al. (2015) observed enhanced consolidation (offline gains) after a single session of anodal right PFC tDCS but no benefit of acquisition (online gains) during implicit sequence learning in young healthy adults. Using a similar implicit learning task, Greeley and Seidler (2019) found that anodal stimulation to left PFC, but not right PFC, facilitated motor learning in young adults, but only when participants remained unaware of the sequence. More recently using the Discrete Sequence Production (DSP) task, an explicit sequence learning paradigm, we observed that either anodal tDCS over the left or right PFC, and cathodal tDCS over the left PFC during practice impaired sequence learning over the course of 3 days in young adults (Greeley et al., 2020). In contrast to using anodal (excitatory) stimulation over the PFC, two studies have observed learning benefits when the PFC is *inhibited* in young adults. Using rTMS, Galea et al. (2010) found enhanced retention after either the left or right dorsolateral PFC was disrupted immediately after learning. Similarly, the application of cathodal (inhibitory) tDCS over the left PFC resulted in increased golf putting performance relative to the sham group (Zhu et al., 2015). The results from Galea et al. (2010) and Zhu et al. (2015) suggest that engaging the prefrontal cortices during or after motor learning may impair learning for young adults. However, older adults consistently recruit frontal regions when performing a range of motor learning tasks (Wu and Hallett, 2005; Lin et al., 2012; Michely et al., 2018). This greater recruitment of prefrontal brain regions during motor task performance has been suggested to serve a compensatory role for older adults (cf. Seidler et al., 2010). Specifically, inhibitory non-invasive brain stimulation applied over brain regions thought to be involved in compensatory processes disrupts performance for older but not young adults (Rossi et al., 2004; Zimerman et al., 2014). Therefore, it may be that excitatory PFC stimulation aids motor learning in older adults. Thus, we sought to understand how using anodal tDCS over left or right PFC would affect learning and retention of a motor sequence in older adults.

We evaluate motor learning here using indices of reaction time and sequence chunking. Chunking, or the ability to “chunk” together individual actions, is thought to facilitate motor learning by reducing memory loads. It is evidenced by a significant difference between inter-response times of key presses (Verwey and Eikelboom, 2003; Kennerley et al., 2004; Bo et al., 2009). Age-related declines in motor learning may stem from declines in working memory, which limits motor chunking. For example, working memory capacity was positively correlated with motor sequence chunk length in both young and older adults (Bo and Seidler, 2009). Older adults showed significantly reduced working memory capacities and sequence chunk lengths relative to young adults. In addition, 22% of older adults showed no evidence of chunking; this was the case for only 7% of young adults. The link between the prefrontal cortices and working memory is well established (Kane and Engle, 2002; Funahashi, 2017) and it is known that older adults show significant reductions in prefrontal gray matter volume relative to young adults (Scahill et al., 2003; Esiri, 2007). Two fMRI studies have reported that young adults recruit the left dorsolateral PFC and right supplementary motor area (SMA) (Pammi et al., 2012) and the mid-left dorsolateral PFC (Wymbs et al., 2012) during sequence chunking. Moreover, Verwey et al. (2019) showed that unfamiliar sequences activated the PFC whereas familiar sequences did not; they instead elicited SMA activation. Thus, it is possible that targeting prefrontal regions (which have been associated with chunking) with anodal tDCS may facilitate motor sequence learning in older adults.

In the current study, we sought to understand the contributions of the left and right prefrontal cortices to motor sequence learning and chunking in healthy older adults; participants received stimulation for 2 days and performance was assessed over 3 days. We tested four different anodal tDCS groups in which either the left PFC, the right PFC, the left M1, or the SMA were stimulated in two separate sessions and compared with a sham group to understand how excitatory stimulation affects motor performance and learning (using the right hand) over the course of three separate sessions. Specifically, we sought to understand whether anodal tDCS to the right or left PFC would enhance or interfere with sequence learning as assessed by reaction times and chunking in a *DSP* task as assessed by the correlation between key presses (Acuna et al., 2014), in older adults. We hypothesized that regardless of the hemisphere of stimulation, older adults would display motor learning benefits in both reaction time and chunking across sequence learning with prefrontal tDCS. Similar to previous tDCS results (Zimerman et al., 2013; Dumel et al., 2016; Greeley et al., 2020), we hypothesized that regardless of age, the M1 tDCS groups would display faster learning as measured by reaction time and number of chunks and given its role in chunking, we hypothesized that the SMA tDCS older adult group would display faster learning as measured by the number of chunks; we also expected that older adults would show greater benefits due to having more room for improvement.

## Materials and Methods

### Participants

We recruited sixty-three older adult participants (age range 64–84, mean age = 70.7, ± 5.76 years; 29 male) and sixty-four young adult participants (age range 18–30, mean age = 20.5, ± 2.4 years, 26 male) from the University of Michigan campus and greater Ann Arbor area. It should be noted that the young adult data has been previously published (Greeley et al., 2020), and the main focus of the current paper is on older adults. We include the young adult data in some of the analyses here to allow for age group comparisons when appropriate. All participants were right handed as measured by the Edinburg handedness inventory (Oldfield, 1971), and reported no history of mental health events, drug abuse, neurological, or psychiatric disorders. During the first session, all participants signed a consent form approved by the University of Michigan Institutional Review Board, verbally answered an alcohol and drug abuse screening questionnaire, completed the Beck Depression inventory (Beck et al., 1988), a custom tDCS screening form, and the Montreal Cognitive Assessment (Nasreddine et al., 2005). Participants were excluded if they scored <23 on the MOCA (Lee et al., 2008; Carson et al., 2018), had a self-reported history of alcohol or drug abuse, and scored >13 on the Beck Depression Inventory. Additionally, participants were not taking medications that could interact with the central nervous system. Participants were compensated for their time at an hourly rate of $15 per hour.

### Discrete Sequence Production Task

**FIGURE 1:**
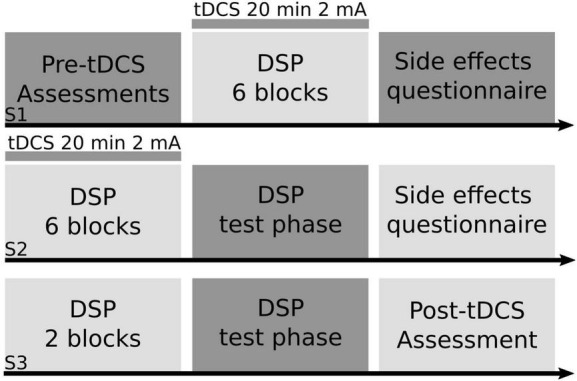
Study design. Using a between-subjects design, right-handed older adult participants practiced a motor sequence task paired with transcranial direct current stimulation (tDCS). Participants were randomly assigned into one of four anodal tDCS groups (right prefrontal cortex, left prefrontal cortex, left primary motor cortex, supplementary motor complex) or sham for the entirety of the study. In session one (S1) older adult participants completed a battery of cognitive and motor tasks (pre-tDCS assessments), followed by six blocks of the discrete sequence production (DSP) task paired with either real or sham tDCS for a maximum of 20 min at 2 mA, followed by a tDCS side effects questionnaire. In session two (S2), participants practiced another six blocks of the DSP task paired with either real or sham tDCS, followed by a test portion of the DSP task, where participant’s explicit knowledge of the sequence was tested, followed by a tDCS side effects questionnaire. In session three (S3), participants completed two blocks of the DSP task without tDCS, then completed the test portion of the DSP task, followed by the same battery of cognitive and motor tasks (post-tDCS assessment) that participants completed at the start of S1.

The study design implemented in the current paper was identical to that used in our recent publication (Greeley et al., 2020). See Figure 1 for an abbreviated overview. Participants practiced a variant of the *DSP* task (Ruitenberg et al., 2014) programmed and presented in E-Prime (version 2.0) over the course of three sessions, two of which participants received either real or sham tDCS, while using their dominant, right hand to practice the sequence task. Participants were assigned two, six-element sequences for the duration of the study. One of the sequences had a repeated, simple structure (e.g., NCBNCB) whereas the other sequence did not have a structure and was thus considered complex (e.g., BCVNVC). Participants could practice each sequence up to a total of 224 times throughout the three sessions of practice (16 times per block across 14 blocks). To simplify data presentation and to be consistent with our previously published results (Greeley et al., 2020), we limited our analysis to the complex sequences. During sequence practice, participants placed their index, middle, ring, and pinky fingers of their dominant, right hand on the C, V, B, and N keys of a keyboard, respectively. Four, horizontally aligned white squares with black trim were presented in the middle of a monitor with a white background. During practice, one of the squares was filled in by a light green color for up to 2,000 ms, which cued participants to press the spatially corresponding key as quickly and accurately as possible. If the participant pressed the correct key, the green square returned to white and the next square in the sequence would immediately turn green. If participants made an incorrect key press, the message “mistake, again” was displayed on the screen in red for 1,000 ms. If participants did not respond to a stimulus within 2,000 ms of the message, “no response, again” was displayed at the bottom of the screen for 1,000 ms. Participants practiced their sequences across 3 days, with six blocks of practice during session one, another six blocks of practice during session two, and two blocks of practice in session three. Participants practiced each of their two sequences eight times during each block of practice. During sessions one and two when participants received tDCS, participants practiced their assigned sequences a total of 96 times.

Participants also received feedback halfway through a block of practice (after 8 of the 16 trials). The feedback screen was shown for 10 sec and displayed each participant’s error percentage and mean reaction time. After 10 sec had passed, participants immediately started on the second half of the block. After completing an entire block of practice, another feedback screen was shown for 50 sec. This feedback screen had the same information as the mid-block feedback screen, however, at the bottom of the screen there was text which read, “After this, practice block x will start.” Before each block (excluding block 1) on practice sessions one and two, the screen displayed, “As you have noticed, there are 2 fixed sequences. Please learn them! We will continue with the same task.”

In sessions two and three immediately after the *DSP* awareness questionnaire (see below), participants completed an additional test phase of the *DSP* task, which consisted of four conditions. Each condition comprised 48 trials (24 of each sequence) and followed the same structure as practice. In the *familiar* condition, the stimuli were presented in the same way as during practice. In the *single-stimulus* condition, only the first stimulus of the sequence was presented as a green square. Once the correct key was pressed, the rest of the sequence was to be completed by the participants without the help of the squares turning green. In the *mixed-familiar* condition, 75% of the trials had modifications to the sequences. The modifications were that two of the six stimuli were changed. In the *unfamiliar* condition, participants were exposed to two sequences that they had not previously experienced.

### Transcranial Direct Current Stimulation Set-Up

**FIGURE 2:**
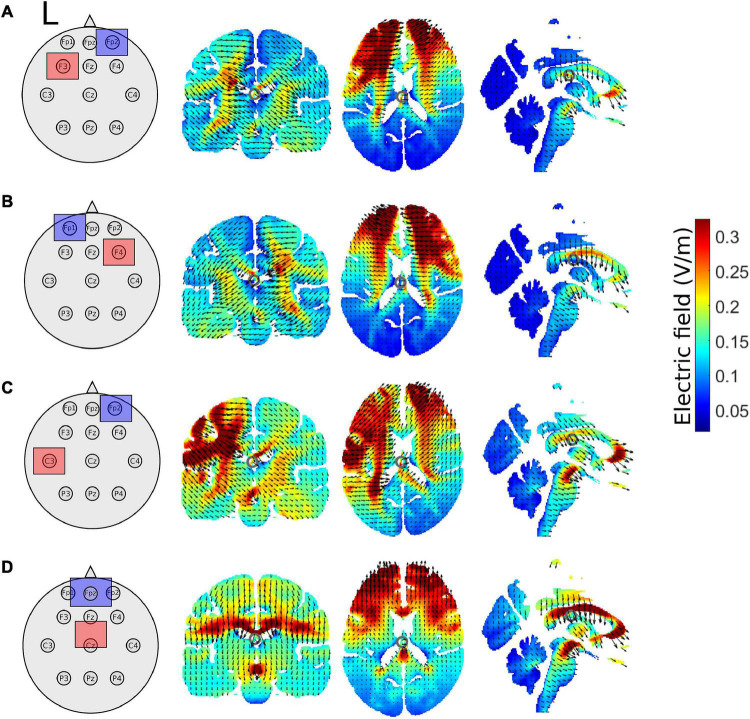
Schematic of tDCS electrode montage and electric field magnitude distribution with current arrows in the coronal, axial, and sagittal slices for the **(A)** left prefrontal montage, **(B)** right prefrontal montage, **(C)** the left M1 montage, and **(D)** for the supplementary motor complex montage produced by the ROAST model. All images are in *x* = –1, *y* = –17, and *z* = 17 MNI space. The left hemisphere is denoted by L. Red squares represent the anode, whereas blue squares represent the cathode.

Participants were randomly assigned into one of five tDCS groups. tDCS was only applied during sessions one and two. All electrode placements were according to the 10–20 EEG system. If participants were randomized into the right or left prefrontal tDCS groups, the anode was placed over the scalp location F4 or F3, respectively and the reference/cathode electrode was placed over the contralateral orbit. For the left M1 stimulation group, the anode was placed over the C3 location and the cathode was placed over the contralateral orbit. For the placement of the anode electrode in the SMA region (henceforth referred to as the supplementary motor complex (SMC) group), we placed the anode 8.7% of the measured distance between the nasion and inion anterior to Cz (approximately 3 cm anterior to Cz) with the cathode over Fpz. The sham tDCS group received the same montage as the real, M1 tDCS group (anode over C3, cathode over contralateral orbit; Figure 2). Stimulation was always 2 mA based on safety recommendations (Iyer et al., 2005; Bikson et al., 2009), previous reports inducing plasticity in young (Monte-Silva et al., 2013; Waters-Metenier et al., 2014; Seidler et al., 2017; Waters et al., 2017; Greeley and Seidler, 2019; Greeley et al., 2020) and older adults (Dumel et al., 2016; Nomura and Kirimoto, 2018; Farnad et al., 2021), and recent evidence suggesting 2 mA is necessary to achieve a physiologically meaningful effect on the cortex (Huang et al., 2017; Filmer et al., 2020). We applied stimulation using a conventional tDCS device (Soterix Medical Inc., New York, NY, United States) for a maximum of 20 min *via* two rubber electrodes placed inside saline soaked sponges. Electrode size was always 5 × 5 cm, except for the SMC montage in which case the anode was 5 × 5 cm and the cathode was 5 × 7 cm (Vollmann et al., 2013); it is unlikely that this larger cathodal electrode comprises tDCS-induced corticospinal excitability changes (Nitsche et al., 2007). Setup for tDCS was the same for sessions one and two. tDCS was not administered during session three.

### Realistic Volumetric-Approach-Based Simulator for Transcranial Electrical Stimulation Model

We used realistic volumetric-approach-based simulator for transcranial electrical stimulation (ROAST) (version 3.0), or Realistic vOlumetric-Approach to Simulate Transcranial electric stimulation, an open source tool (Huang et al., 2019). The output from the ROAST model was used as a qualitative means to aid in the interpretations of the electric field distributions of our electrode montages. We ran the ROAST model a total of 4 times for each tDCS electrode montage, electrode locations used in the model are in parentheses. The output of the model can be observed for the left PFC anode (F3), right orbitofrontal cortex cathode montage (Fp2; Figure 2A), the right PFC anode (F4), left orbitofrontal cortex cathode montage (Fp1; Figure 2B), the left M1 anode (C3), right orbitofrontal cortex cathode montage (Fp2; Figure 2C), and the SMA complex anode (FCz), orbit cathode montage (Fpz; Figure 2D). Simulations were run using the MNI152 averaged head. We specified pad electrodes as the electrode type with a height of 3 mm.

## Procedure

During session one, as part of the pre-tDCS assessments, participants completed Thurstone’s card rotation task (Ekstrom et al., 1979), a custom computerized version of the visual search task, the digit symbol substitution task (Wechsler, 1958), a modified version of the visual array change working memory assessment (Luck and Vogel, 1997), three trials of the Purdue pegboard task (Tiffin and Asher, 1948) and a grip strength assessment before *DSP* practice. After this neuropsychological assessments, participants took a mandatory 3–5 min break before tDCS setup and *DSP* practice. After setup, we turned stimulation up to 1 mA (pre-stimulation tickle) for 15 s to ensure satisfactory contact quality of the electrodes on the scalp. After 15 s of stimulation, participants completed a shortened 10-item Positive and Negative Affect Schedule (PANAS) mood inventory. Then the participants were instructed on the *DSP* task. After the instructions, the experimenter would start the stimulation and the tDCS unit would ramp up over a period of 30 sec to full intensity (always 2 mA) and then remain active up to 20 min in duration. After the 6 blocks of practice, tDCS was turned off (for active tDCS) only if participants completed practice under 20 min (*n* = 7). For reference, participants took 23.3 min (± 3 min) to complete practice in session one. The right PFC group took 23.9 min (± 2.3), the left PFC group took 23.2 min (± 3.6), the left M1 group took 22.2 min (± 2.8), the SMC group took 23.6 min (± 3.5) and the sham group took 23.7 min (± 3.3). There were no group differences in terms of the amount of time it took to complete the task in session one. For the sham tDCS group, the tDCS would stay on for 30 sec then ramp back down to 0 mA. If the task was not completed by the 20 min timer built into the tDCS unit, stimulation was automatically stopped by the device. Immediately following the 6 blocks of *DSP* practice, we administered a second version of the digit symbol coding task, the PANAS mood survey, and a custom tDCS side effects questionnaire. After the participant completed the tDCS side effects questionnaire, we removed the electrodes and sent the participants home with physical exercise and handedness questionnaires (Oldfield, 1971) to complete and return at their next visit (exercise data not included here).

Session two took place following at least one night’s sleep but no longer than 72 h after session one. All but nine participants came to session two within 24 h of session one, whereas one participant came to session two within 72 h and eight participants came to session two within 48 h of session one (*M* = 1.2 days). In session two, participants completed the card rotation task followed by the digit symbol substitution task. tDCS was setup similar to session one, then pre-stimulation tickle was administered to ensure satisfactory contact quality. After the pre-stimulation tickle, we administered the mood survey and summarized the instructions of the *DSP* task. If participants had no questions, we turned on the tDCS, let the unit ramp up to full intensity, and started the *DSP* task (blocks 7–12, totaling 96 trials per sequence after session 2 at this point). For sham, the stimulation would again stay on 30 sec then ramp back down to 0 mA. Immediately following practice, the *DSP* awareness questionnaire was administered (tDCS stimulation was off at this point). The questionnaire was followed by instructions of the test portion of the *DSP* task. After the test portion of the task, participants completed the digit symbol substitution coding task and the mood survey again, and finally the tDCS side effects questionnaire.

The third session commenced at least one night’s sleep but no longer than 72 h after session two. All but nine participants came into session three within 48 h of session two (*M* = 1.2 days). The main purpose of session three was to measure the impact of stimulation on the sequences (i.e., testing conditions) in the absence of stimulation. In session three, participants completed two blocks of practice (blocks 13–14, totaling 112 practice trials per sequence), the *DSP* awareness questionnaire, then the test portion of the *DSP* task. Participants were offered a break, then completed the battery of post-tDCS intervention assessments including the card rotation test, the visual search task, the digit symbol substitution coding task, and the visual array change task. Lastly, participants completed an exit survey to probe for strategies used during practice.

### Discrete Sequence Production Awareness Questionnaire

Immediately following practice on sessions two and three, participants were asked about their awareness of the sequences. The first two questions probed the participants’ knowledge of the questions by asking them to write down the two sequences they had practiced. The second two questions asked participants to verbally tell the experimenter what the sequences were from memory. The third question required participants to choose two sequences from a list of eighteen possible sequences. The *DSP* awareness questionnaire took approximately 5 min to complete.

### Data Analyses

Data are presented as mean ± standard deviation (SD) unless otherwise noted. To study motor learning, our primary outcomes were reaction time, number of (motor) chunks, and number of errors for the complex sequences across the 3 days of practice. Prior to statistical analysis, data were checked to determine whether they were normally distributed. We opted to use a linear mixed model to investigate the effect of stimulation on the *rate* of learning across practice trials given the reaction time data for the older adults were partially skewed and linear mixed models can handle this data well (Arnau et al., 2012; Lo and Andrews, 2015; Schielzeth et al., 2020). Additionally, linear mixed models can handle missing data; every participant had a different number of removed trials due to errors. We also implemented separate ANOVAs to investigate the effect of stimulation on the testing conditions.

Using the software Stata (version 13.0), two linear mixed models were implemented using reaction time and number of chunks as dependent variables limited to the older adult group. Trials were used as a continuous factor, whereas Stimulation Group and Session were used as blocked factors. Random intercepts and fixed slopes were used for each participant. If a significant main effect or interaction emerged within the older adult group, follow-up pairwise comparisons were used to determine which pairs of the factor levels are significantly different from each other, always comparing each real stimulation group to sham if applicable. Additionally, if a significant difference between any active tDCS (right PFC, left PFC, left M1, SMC) and sham tDCS group emerged within the older adult group, we then included the young healthy adults receiving the same electrode montages in a follow-up analysis to understand whether tDCS differentially affected the age groups. *P*-values and confidence intervals were adjusted within Stata using Scheffé’s method for pairwise comparisons. Prior to statistical analyses, data were checked to satisfy normality. To calculate an effect size for each pairwise comparison, we used the esize function in Stata, using number of observations, mean, and standard deviation to obtain Cohen’s d (*d*). A computational model developed by Acuna et al. (2014) was used to determine the number of chunks, which uses reaction times as well as the covariation across key presses to detect chunk boundaries. In contrast to the linear mixed model which allowed us to understand how stimulation may affect the *rate* in learning, one-way ANOVAs were used to test whether there were any overall benefits of real tDCS relative to sham within the older adult group in terms of performance (magnitude). Independent *t*-tests were used to test for baseline demographic differences between the two age groups in terms of the Purdue Pegboard, visual array capacity, and MoCA. We also completed a series of independent *t*-tests and Mann-Whitney *U* tests to check for baseline demographic differences between the real tDCS and sham groups within older adults. For the number of errors, we first used a Kruskal–Wallis test to compare young and older adults, then a series of Mann–Whitney tests comparing each active tDCS group to sham within each session limited to the older adult group (critical a’ = 0.004). We also ran an additional 2 (Age group: young, old) by 5 (tDCS group: right PFC, left PFC, M1, SMC, sham) by 2 (Session: 2, 3) by 4 (Testing Condition: familiar, mixed familiar, mixed unfamiliar, and single stimulus) repeated measures ANOVA on the reaction time of the testing phase of the *DSP* task (see *DSP* description above).

## Results

Two older adults were unable to return after session one and three older adult participants were unable to return after session two. An additional two older adult participants were removed from analysis due to exessive errors (>3 SD). The breakdown of final sample sizes by stimulation group were the following: the right PFC (*n* = 13), left PFC (*n* = 12), left M1 (*n* = 12), SMC (*n* = 12), and sham (*n* = 12).

### Demographics

Independent *t*-tests revealed demographic differences between the old and young adult groups. Young adults displayed higher MOCA scores [*t*(121) = 4.04, *p* < 0.001] and better manual function for the left [*t*(115) = 8.10, *p* < 0.001] and right [*t*(115) = 9.65, *p* < 0.001] hand as indicated by the number of pegs completed in the Purdue Pegboard test. Visuospatial working memory was also statistically different in that older adults showed an overall lower capacity [*t*(121) = 8.69, *p* < 0.001]. See Table 1 for group averages. There were no demographic differences between any of the real tDCS and sham groups (*p’s* > 0.37). See Table 2 for older adult group demographic averages. Additionally, no older adults reported having experienced adverse effects (Supplementary Table 1).

**TABLE 1:** Mean (SD) age group scores for the MoCA, Purdue Pegboard, and spatial working memory capacity (visual array capacity).

	YA group	OA group
	**(*N* = 64)**	**(*N* = 63)**
MoCA	28.42 (1.51)	27.16 (1.92)*
VAC capacity	4.67 (1.0)	3.00 (1.1)*
Purdue right	16.11 (1.73)	13.0 (1.76)*
Purdue left	14.84 (1.62)	12.37 (1.67)*

MoCA, Montreal cognitive assessment; OA, older adults; VAC, visual array capacity; YA, young adults. *p < 0.05.

**TABLE 2:** Mean (SD) transcranial direct current stimulation (tDCS) group scores of age, MoCA, sex, Purdue Pegboard, and spatial working memory capacity (visual array capacity) for older adults.

	OA tDCS group				
	Right PFC (*n* = 13)	Left PFC (*n* = 12)	Left M1 (*n* = 12)	SMC (*n* = 12)	Sham (*n* = 12)
Age	69.2 (5.1)	70.8 (4.7)	72.8 (6.9)	71.0 (6.1)	68.5 (4.5)
MoCA	26.7 (2.3)	26.7 (1.3)	27.6 (0.9)	27.2 (2.4)	27.5 (2.0)
Sex	7 F/6 M	7 F/5 M	6 F/6 M	6 F/6 M	7 F/5 M
VAC capacity	2.9 (1.1)	3.2 (1.1)	2.7 (1.2)	3.2 (1.3)	3.1 (0.7)
Purdue right	13.2 (1.6)	13.1 (1.3)	12.5 (1.9)	13.2 (2.1)	13.1 (2.0)
Purdue left	12.6 (2.0)	12.8 (1.4)	11.8 (1.6)	11.8 (1.7)	12.8 (1.7)

*There were no group differences between any of the real tDCS groups and sham for any of the demographic data (p’s > 0.36). MoCA, Montreal cognitive assessment; OA, older adults; VAC, visual array capacity.*

### Reaction Time

The linear mixed model revealed a main effect of Session [χ*^2^*(2, *N* = 61) = 441.68, *p* < 0.001]. Reaction time in the second session decreased at a lower rate compared to the first session (β = 0.60, SE = 0.03, *p* < 0.001, *d* = −0.61), whereas reaction time decreased at a faster rate in session three relative to session two (β = −0.37, SE = 0.11, *p* = 0.005, *d* = −0.07). There was no main effect of Stimulation Group (χ*^2^*(4, *N* = 61) = 8.76, *p* = 0.067).

**FIGURE 3:**
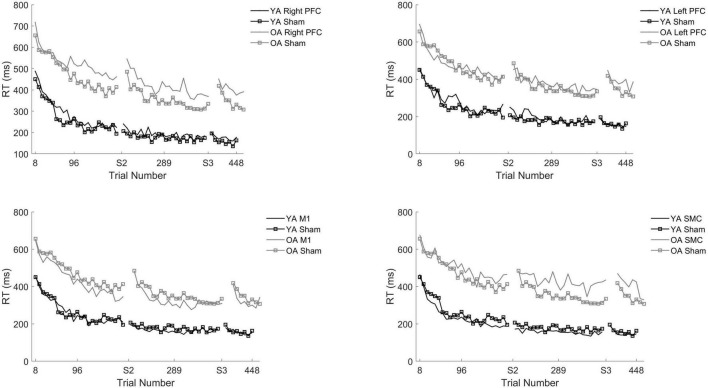
Reaction time (RT) for young and older adults as a function of trial number. RT was binned across every eight trials. Each panel represents a tDCS stimulation location and sham group for reference. **(Top left)** Young (black) and older (gray) adults in the right prefrontal cortex (PFC) tDCS groups as well as young (black with squares) and older (gray with squares) sham groups. **(Top right)** Young (black) and older (gray) adults in the left PFC tDCS groups as well as sham (squares). **(Bottom left)** Young (black) and older (gray) adults primary motor cortex (M1) tDCS groups. **(Bottom right)** Young (black) and older (gray) adults in the supplementary motor complex (SMC) tDCS groups.

**FIGURE 4:**
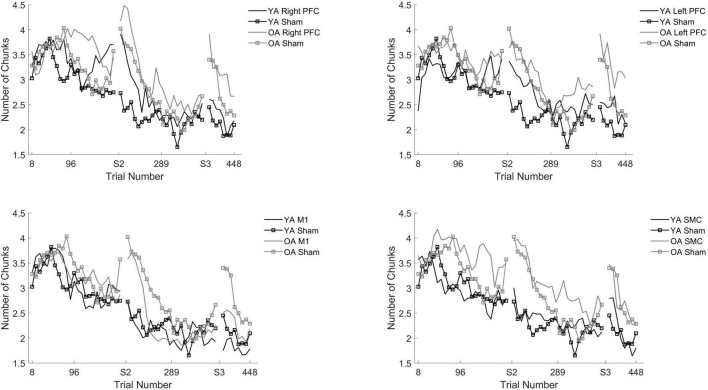
Averaged reaction time for young (black) and older adults (gray) for each tDCS group within each practice session. Each panel represents one session. Error bars represent standard deviation.

There was a Stimulation Group by Session interaction [χ*^2^*(8, *N* = 61) = 65.71, *p* < 0.001]. To understand the interaction, follow-up pairwise contrasts were performed, with Scheffé correction. Older adults in the right PFC (β = 0.39, SE = 0.06, *p* = 0.024, *d* = 0.33) and SMC (β = 0.43, SE = 0.07, *p* = 0.003, *d* = 0.23) tDCS groups decreased their reaction times at a slower rate relative to older adults in the sham group during session one (Figures 3, 4 and Tables 3, 4). No other comparisons reached significance.

**TABLE 3:** Results from linear mixed model using reaction time as the dependent variable.

				Adjusted			
Reaction time	β	Std Err.	Z	*p*-value	95% conf. interval		Cohen’s d
**Session**							
Session 2 vs. Session 1	0.597	0.030	20.160	**<0.001**	0.524	0.669	−0.607
Session 3 vs. Session 2	−0.968	0.114	−8.500	**<0.001**	−1.25	−0.689	−0.050
**Stimulation by session**							
Sham vs. R PFC, session 1	−0.388	0.065	−5.980	**0.001**	−0.705	−0.072	−0.333
Sham vs. L PFC, session 1	−0.296	0.066	−4.460	0.133	−0.619	0.027	
Sham vs. M1, session 1	0.188	0.067	2.830	0.888	−0.135	0.512	
Sham vs. SMC, session 1	−0.431	0.066	−6.490	**<0.001**	−0.755	−0.108	−0.704
Sham vs. R PFC, session 2	0.033	0.067	0.490	1.000	−0.292	0.358	
Sham vs. L PFC, session 2	−0.095	0.066	−1.440	1.000	−0.416	0.227	
Sham vs. M1, session 2	−0.078	0.065	−1.190	1.000	−0.395	0.239	
Sham vs. SMC, session 2	−0.205	0.065	−3.160	0.765	−0.521	0.111	
Sham vs. R PFC, session 3	−0.473	0.354	−1.340	1.000	−2.198	1.251	
Sham vs. L PFC, session 3	−0.409	0.347	−1.180	1.000	−2.100	1.282	
Sham vs. M1, session 3	−0.468	0.351	−1.330	1.000	−2.178	1.242	
Sham vs. SMC, session 3	−0.212	0.344	−0.610	1.000	−1.886	1.463	

*All p-values and confidence intervals have been Scheff adjusted. β, beta; conf., confidence. Std Err., standard error. Significant p-values are in bold.*

**TABLE 4:** Means (SD) of reaction time and number of chunks for each stimulation group, age group, and session.

	Reaction time		Number of chunks	
Session 1	YA	OA	YA	OA
Right PFC	290.20 (77.77)	532.60 (135.65)	3.47 (0.69)	3.62 (0.49)
Left PFC	282.66 (111.75)	477.99 (140.47)	3.10 (0.61)	3.48 (0.64)
M1	269.08 (87.04)	447.38 (152.53)	3.17 (0.40)	3.27 (0.50)
SMC	249.61 (95.72)	513.33 (199.43)	3.05 (0.78)	3.27 (0.50)
Sham	271.92 (117.35)	477.71 (116.69)	3.08 (0.56)	3.41 (0.63)
**Session 2**				
Right PFC	191.28 (60.19)	421.68 (133.84)	2.58 (0.57)	3.02 (0.84)
Left PFC	187.99 (85.12)	375.23 (126.90)	2.65 (0.80)	2.94 (0.75)
M1	166.93 (56.36)	332.96 (173.74)	2.12 (0.66)	2.33 (0.46)
SMC	159.53 (56.87)	437.43 (219.75)	2.33 (0.68)	2.33 (0.46)
Sham	177.12 (97.24)	357.27 (114.34)	2.25 (0.77)	2.73 (0.83)
**Session 3**				
Right PFC	176.10 (53.85)	404.05 (156.47)	2.31 (0.99)	3.11 (0.98)
Left PFC	156.17 (53.30)	393.93 (155.65)	2.38 (0.86)	3.18 (1.52)
M1	154.74 (48.08)	331.46 (186.53)	1.80 (0.62)	2.25 (0.76)
SMC	151.39 (42.43)	413.74 (213.31)	2.14 (0.83)	2.25 (0.76)
Sham	162.54 (90.45)	345.48 (119.28)	2.13 (0.88)	2.84 (1.11)

*M1, primary motor cortex; OA, older adults; PFC, prefrontal cortex; SMC, supplementary motor complex; YA, young adults.*

To understand whether tDCS had differential effects between the young and older adults, we included two additional tests: one limited to young and older adults in the right PFC and sham tDCS groups within session one and another limited to young and older adults in the SMC and sham tDCS groups within session one. For the right PFC and sham tDCS groups, we observed a Stimulation by Age Group interaction [χ*^2^*(1, *N* = 27) = 29.02, *p* < 0.001]. Pairwise comparisons revealed that older adults in the right PFC reduced reaction time at a significantly slower rate relative to young adults in the right PFC tDCS group (β = 0.24, SE = 0.06, *p* = 0.002, *d* = 1.67) (Table 4). Importantly, while older adults in the sham group reduced reaction times at a significantly faster rate compared to older adults in the right PFC tDCS group, there was no difference between the sham and PFC tDCS groups for young adults (*p* = 0.704). Similarly, we observed a Stimulation by Age Group interaction [χ*^2^*(1, *N* = 25) = 24.34, *p* < 0.001] for the SMC and sham tDCS groups. Pairwise comparisons revealed that older adults in the SMC group reduced reaction time at a significantly slower rate relative to young adults (β = 0.19, SE = 0.06, *p* = 0.021, *d* = 1.56) (Table 4). While older adults in the sham group reduced their reaction times at a faster rate compared to older adults in the SMC group, there was no difference between the sham and SMC tDCS groups for young adults (*p* = 0.989).

To understand the overall impact of tDCS within older adults a series of one-way ANOVA contrasts comparing each active tDCS group to sham within each session. No contrasts were significant (*p* > 0.4; Figures 3, 4).

### Number of Chunks

The linear mixed model revealed significant main effects of Stimulation Group [χ *^2^*(4, *N* = 60) = 20.79, *p* < 0.001] and Session [*X*^2^(2, *N* = 60) = 279.33, *p* < 0.001] for older adults.

**FIGURE 5:**
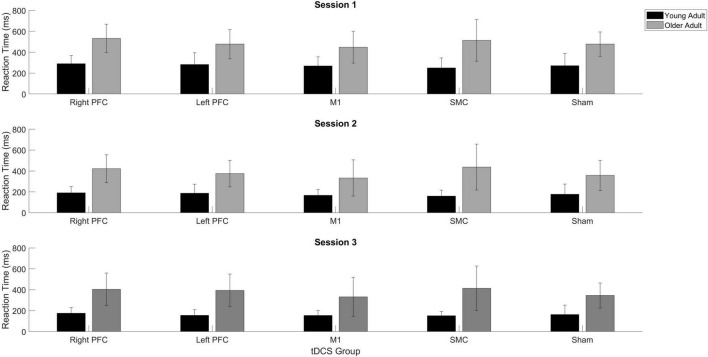
Number of chunks for young and older adults as a function of trial number. Number of chunks were binned across every eight trials. Each panel represents a tDCS stimulation location and sham group for reference. Top left panel depicts young (black) and older (gray) adults in the right prefrontal cortex (PFC) tDCS groups as well as young (black with squares) and older (gray with squares) sham groups. Top right panel depicts young (black) and older (gray) adults in the left PFC tDCS groups as well as sham (squares). Bottom left panel depicts young (black) and older (gray) adults primary motor cortex (M1) tDCS groups. Bottom right panel depicts young (black) and older (gray) adults in the supplementary motor complex (SMC) tDCS groups.

We performed follow-up pairwise comparisons to understand each main effect. We observed a significantly faster rate in the reduction in the number of chunks in session two relative to session one (β = −0.01, SE = 0.00, *p* < 0.001, *d* = −0.61) and a significantly faster rate in the reduction in the number of chunks in session three relative to session two (β = −0.01, SE = 0.00, *p* < 0.001, *d* = 0.03). Across all sessions, the left prefrontal (β = 0.01, SE = 0.00, *p* = 0.002, *d* = 0.19) and the M1 (β = 0.01, SE = 0.00, *p* = 0.023, *d* = −0.23) tDCS groups reduced the number of chunks at a significantly slower rate relative to the sham tDCS group (Figure 5 and Table 5). No other pairwise comparisons reached significance.

**TABLE 5:** Results from linear mixed model using number of chunks as the dependent variable for older adults.

				Adjusted			
Chunking	β	Std Err.	Z	*p*-value	95% conf. interval		Cohen’s d
**Session**							
Session 2 vs. Session 1	−0.005	<0.001	−14.06	**<0.001**	−0.006	−0.004	−0.613
Session 3 vs. Session 2	−0.010	0.001	−7.11	**<0.001**	−0.014	−0.007	0.029
**Stimulation**							
Right PFC vs. Sham	0.004	0.001	2.60	0.150	−0.001	0.001	
Left PFC vs. Sham	0.006	0.002	4.17	**0.002**	0.002	0.012	0.190
M1 vs. Sham	0.005	0.001	−3.37	**0.023**	−0.010	<−0.001	−0.232
SMC vs. Sham	0.003	0.002	1.75	0.546	−0.002	0.007	
**Stimulation by session**							
Right PFC vs. Sham, session 1	0.004	0.001	5.36	**0.011**	<0.001	0.008	0.222
Left PFC vs. Sham, session 1	0.002	0.001	2.84	0.885	−0.002	0.007	
M1 vs. Sham, session 1	<0.001	0.001	0.56	1.000	−0.004	0.004	
SMC vs. Sham, session 1	0.002	0.001	2.14	0.991	−0.002	0.006	
Right PFC vs. Sham, session 2	−0.001	0.001	−0.95	1.000	−0.005	0.003	
Left PFC vs. Sham, session 2	0.005	0.001	5.39	**0.010**	<0.001	0.009	0.201
M1 vs. Sham, session 2	0.002	0.001	2.38	0.975	−0.006	0.002	
SMC vs. Sham, session 2	0.001	0.001	0.78	1.000	−0.003	0.005	
Right PFC vs. Sham, session 3	0.008	0.004	1.85	0.998	−0.013	0.028	
Left PFC vs. Sham, session 3	0.012	0.004	2.74	0.912	−0.009	0.033	
M1 vs. Sham, session 3	0.013	0.004	−2.94	0.853	−0.034	0.008	
SMC vs. Sham, session 3	−0.005	0.004	1.25	1.000	−0.016	0.027	

*All p-values and confidence intervals have been Scheffé adjusted. β, beta; conf., confidence. Std Err., standard error. Significant p-values are in bold.*

The model also revealed a Stimulation Group by Session interaction [χ*^2^*(8, *N* = 60) = 53.93, *p* < 0.001]. Pairwise comparisons revealed that older adults in the right prefrontal tDCS group (β = 0.00, SE = 0.00, *p* = 0.011, *d* = 0.22) reduced the number of chunks at a slower rate across trials compared to sham in session one. Similarly, older adults in the left prefrontal tDCS group (β = 0.00, SE = 0.00, *p* = 0.010, *d* = 0.20) reduced the number of chunks at a slower rate across trials than sham in session two. No other pairwise comparisons reached significance.

To understand whether the impairment of right or left prefrontal anodal tDCS on the rate of reduction in the number of chunks was different across age groups, we ran two additional tests. The first test included both young and older adults limited to the right prefrontal and sham tDCS groups within session one. We observed that there was no Stimulation by Age Group interaction [*X*^2^(1, *N* = 26) = 1.34, *p* = 0.247]. The second test included both young and older adults in the left prefrontal and sham tDCS groups within session two. We observed there was a significant Stimulation by Age Group interaction [*X*^2^(1, *N* = 26) = 54.65, *p* < 0.001]. The difference between the left prefrontal and sham tDCS groups was larger for the young than the older adults. That is, in session two left prefrontal stimulation was associated with more chunks for the young than the older adults, relative to sham (Figure 5 and Table 4).

To understand the overall impact of tDCS within OA we completed three, one-way ANOVA contrasts comparing each active tDCS group to sham within each session. There was a significant main effect limited to Session [*F*(4, 58) = 2.648, *p* = 0.043]. Planned contrasts showed no differences between the active tDCS groups and sham (*p* > 0.1) (Table 4).

### Errors

Within the older adult group, there were no differences in error commissions between any of the real and sham tDCS groups. Therefore, we performed three separate Mann–Whitney *U* tests to determine whether there was an age group effect for the number of errors committed in each session. There was no age group difference in the number of committed errors in session one (*Z* = −1.726, *p* = 0.084), two (*Z* = −1.362, *p* = 0.173), or three (*Z* = −0.317, *p* = 0.751). Within the older adult group, a Wilcoxon Signed Ranks test revealed that older adults reduced the number of errors made between sessions one (*M* = 7.41, SD ± 6.06) and two (*M* = 6.15, SD ± 4.33) (*Z* = −2.63, *p* = 0.009), and between sessions two and session three (*M* = 1.86, SD ± 1.60) (*Z* = −6.214, *p* < 0.001).

In summary, older adult individuals who received tDCS over either the right PFC or SMC showed slowed reaction time. This impairment was greater for older adults than young adults. For the number of chunks, older adults who received either left or right prefrontal tDCS displayed slower chunking. However, both older and young adults were impaired to the same extent.

## Test Conditions

A 2 (Age group: young, old) by 5 (tDCS group: right PFC, left PFC, M1, SMC, sham) by 2 (Session: 2, 3) by 4 (Testing Condition: familiar, mixed familiar, mixed unfamiliar, and single stimulus) repeated measures ANOVA revealed a main effect of Session [*F*(1,86) = 43.9, *p* < 0.001], a main effect of Test Condition [*F*(3,258) = 1717.212, *p* < 0.001], a main effect of Age Group [*F*(1,86) = 90.319, *p* < 0.001], and a main effect of Stimulation Group [*F*(1,86) = 2.985, *p* = 0.023]. Pairwise comparisons revealed that reaction time was significantly slower in session two (*M* = 359 ms, SE ± 6.8 ms) relative to session three (*M* = 342 ms, SE ± 6.7 ms; *p* < 0.001). Reaction time in the familiar test condition (*M* = 222 ms, SE ± 7.8 ms) was shorter compared to the single stimulus (*M* = 236 ms, SE ± 8.6 ms; *p* < 0.001), the mixed (*M* = 468 ms, SE ± 6 ms; *p* < 0.001) and the mixed unfamiliar (*M* = 477 ms, SE ± 6 ms; *p* < 0.001) testing conditions. Older adults had overall longer reaction times (*M* = 414 ms, SE ± 11 ms) relative to the young group (*M* = 287 ms, SE ± 8 ms; *p* < 0.001). There were no significant differences between any real tDCS group and sham.

There was one significant interaction between Test Condition and Age Group [*F*(3,86) = 2.985, *p* = 0.023]. No other interactions were statistically significant (*p* > 0.20). To understand the significant Testing Condition and Age Group interaction, we performed four independent samples *t*-tests, comparing the reaction time within each testing condition between the two age groups. Young adults had shorter reaction times in the familiar condition (*M* = 168 ms, ± SD = 61 ms) relative to the older adults (*M* = 364 ms, ± SD = 160 ms; *t*(240) = −12.762, *p* < 0.001). Young adults also had shorter reaction times in the mixed condition (*M* = 398 ms, ± SD = 55 ms) relative to the older adults (*M* = 560 ms ± SD = 88 ms; *t*(241) = −17.316, *p* < 0.001), faster reaction times in the mixed unfamiliar condition (*M* = 413 ms, ± SD = 52 ms) compared to the older adults (*M* = 568 ms ± SD = 93 ms; *t*(242) = −16.027, *p* < 0.001), and faster reaction times in the single stimulus condition (*M* = 181 ms ± SD = 78 ms) relative to the older adults (*M* = 312 ms ± SD = 126 ms; *t*(208) = −9.285, *p* < 0.001).

## Discussion

In contrast to our hypotheses, multi-session tDCS did not improve motor learning for older adults. Unexpectedly, anodal right prefrontal and SMC stimulation impaired learning for older adults as evidenced by longer reaction times across trials in session one. Similarly, anodal tDCS over either the right or left PFC impaired learning as assessed by the slowed reduction in the number of chunks in sessions one and two, respectively. In contrast to previously reported findings, older adults that received two separate sessions of stimulation to M1 showed no advantage or impairment in learning relative to sham.

Multiple sessions of stimulation over the prefrontal cortices did not enhance motor learning for older adults. In contrast, stimulation to the right PFC impaired learning as evidenced by a slowing of reaction time as well as a slowing in the reduction in the number of chunks for older adults limited to session one and a slowing in the reduction of the number of chunks limited to the left PFC tDCS group within session two. This replicates our previously reported results where we observed that young adults who received anodal (excitatory) tDCS either over the right or left PFC displayed impaired learning in the same motor learning task (Greeley et al., 2020). Based on the consistent impairment of motor sequence learning in young and older adults who received anodal tDCS over the prefrontal cortices, it is possible that using inhibitory stimulation may produce the opposite behavioral results and may be beneficial to motor learning. As such, there are two previous reported examples of non-invasive brain stimulation studies that demonstrate inhibiting the prefrontal cortices may facilitate sequence learning (Galea et al., 2010; Zhu et al., 2015). The findings from these studies suggest that suppressing the declarative memory system promotes the automatization of sequence learning regardless of age.

Counterintuitively, our findings also suggest that inhibiting prefrontal regions in older adults may also promote motor learning. We observed that right prefrontal anodal tDCS impaired learning as measured by both reaction time and the number of chunks limited to session one. Using the same design, we previously reported that anodal tDCS over the right PFC also impaired learning for young adults in session one (Greeley et al., 2020), indicating that the right PFC is involved for both age groups. However, it is of importance to note that when compared to young adults, right prefrontal stimulation was especially harmful to early learning (session one) to older adults as indexed by slowed reaction time. This suggests an integral role of the right PFC in this task and potentially to early motor sequence learning in general, because if the right PFC was not involved in this task stimulating this brain region, in theory, should *not* affect behavior. Our results support the model of hemispheric asymmetry reduction in older adults, or HAROLD (Cabeza, 2002; Przybyla et al., 2011), which suggests that during aging there is an increase in bilateral activity relative to young adults which is thought to be compensatory. While we observed impaired learning in the right prefrontal tDCS group, we found no evidence for an early motor learning impairment for the left prefrontal tDCS older adult group. Thus, it is likely that older adults recruit both the left and right prefrontal cortices during learning and while right prefrontal anodal tDCS is especially harmful to explicit motor sequence learning, the left PFC is able to compensate and contribute to the task while the right hemisphere is affected with the anodal tDCS in the older adult group, whereas young adults display a far greater deficit (Greeley et al., 2020). Taken together, this suggests that regardless of age, tDCS over the right prefrontal cortices impairs learning, however, older adults are able to compensate due to bilateral compensatory mechanisms that are often observed in aging.

Stimulation to the prefrontal cortices in the older adult group also impaired chunking. In the current study we used a computational model that uses the covariation across key presses in order to detect chunk boundaries (Acuna et al., 2014). Using the number of chunks as identified by the Acuna et al. (2014) model, we observed a slower rate of chunking in the right prefrontal group limited to session one and slowed chunking in the left prefrontal group limited to session two. Unlike reaction time, however, we observed that this impairment was no different between the two age groups. Impaired chunking in the left and right PFC tDCS groups in older adults is surprising considering the involvement of the prefrontal cortices during chunking (Pammi et al., 2012; Wymbs et al., 2012), the positive relationship between working memory and chunking in older adults (Bo et al., 2009), and our previously reported findings demonstrating that prefrontal tDCS impaired chunking in young adults (Greeley et al., 2020). It remains an open question, however, whether using inhibitory stimulation (cathodal) over the prefrontal cortices would enhance chunking and whether it would affect older and young adults similarly. Future studies are needed to test these specific hypotheses but seem promising given the current results.

Our results also provide additional insight regarding the differential roles and specific time courses the prefrontal cortices play in motor learning. It is well established that the prefrontal cortices play an integral role early in motor sequence learning (Doyon and Benali, 2005; Floyer-Lea and Matthews, 2005). Our results replicate and extend these finding as we observed that stimulation to the right PFC impaired motor learning as evidenced by both a slower reduction in reaction time and the number of chunks limited to session one. However, we also observed that stimulation to the left, but not the right, PFC impaired chunking in session two. Two neuroimaging studies have also reported a specific role of the left PFC in chunking (Pammi et al., 2012; Wymbs et al., 2012). Taken together, our results suggest that the right PFC is involved early in sequence learning, whereas there is a specific role of the left PFC later in sequence learning related to chunking.

A limitation of the preceding discussion is that it assumes that anodal stimulation has excitatory effects on the underlying cortex. This has been shown in the motor cortex, where anodal stimulation over M1 leads to enhanced motor evoked potentials. However, tDCS electrode montages that do not include M1 have elicited unexpected effects. For instance, placing the anode over F3 and the cathode over F4 results in bilateral cortical excitation, based on functional connectivity changes (Nissim et al., 2019). The results of the current stimulation modeled in ROAST based on our prefrontal electrode montages also suggest a complex pattern of current distribution. The resulting current distribution is similar between our left and right prefrontal stimulation montages, making it less surprising that the two groups would show similar behavioral effects. In our prior work with young adults, we found that both anodal and cathodal stimulation applied to the left PFC resulted in slower sequence learning (Greeley et al., 2020), further supporting the complex effects arising with prefrontal stimulation. Regardless, it is important to know for the future design of potential therapies combining brain stimulation with training that both young and older adults exhibit sequence learning impairments with prefrontal tDCS.

Older adults that received stimulation to the left M1 showed no advantage in motor learning. This finding is in contrast to our previously reported findings in young adults, where we observed a benefit of left M1 stimulation in both reaction time and number of chunks in the same task (Greeley et al., 2020). Several other studies also report that anodal M1 tDCS facilitates learning across a variety of motor sequence tasks in both young (Reis et al., 2009; Stagg et al., 2011; Saucedo Marquez et al., 2013) and older adults (Zimerman et al., 2013; Dumel et al., 2016). Unexpected age-related responses to tDCS such as priming or preconditioning may account for the attenuated and null results observed in the older adults here. For example, older adult participants that received anodal tDCS to M1 immediately, but not an hour or 2 h following training on a motor sequence task, showed enhanced consolidation during a retest 24 h later (Rumpf et al., 2017). Another example comes from Fujiyama et al. (2017) who found enhanced force control and increased corticospinal excitability following a preconditioning period where cathodal (inhibitory) stimulation was applied prior to learning, followed by anodal stimulation during learning. The findings of Rumpf et al. (2017) and Fujiyama et al. (2017) conflict with Stagg et al. (2011), who found that the optimal timing of stimulation in young adults was during, but not before or after, a motor sequence task (Stagg et al., 2011). Thus, the lack of anodal M1 tDCS on motor sequence learning in older adults in the current study may be due to the timing of stimulation administration. Specifically, it may be that stimulating the prefrontal cortices during sequence learning was not an ideal protocol for older adults and instead tDCS should have been performed immediately before or after learning. Future studies should consider exploring how various tDCS timing protocols affect excitability and learning in older adults, as most previous permutations has been exclusively studied in young adults.

The present study is not without limitations. The sample size for each tDCS group is modest; however, the study design required a substantial time commitment from participants. Future studies should consider recruiting larger sample sizes to understand whether the negative impact of anodal PFC tDCS on motor learning can be replicated. Another potential limitation is the task specific effects of tDCS (Saucedo Marquez et al., 2013; Kimura et al., 2021). It is possible that the motor sequencing task employed here is not appropriate to pair with anodal prefrontal stimulation. Not collecting baseline reaction times can also be a potential limitation. However, the inclusion of a sham group similar in age, sex, MoCA, handedness, and time between sessions to the other real stimulation groups helped control for this limitation. While we took several measures to ensure participants were engaged throughout the task such as stretch and water breaks, performance feedback, a relatively short amount of task practice time each session (∼20 min), and monetary compensation, it is possible that participants could have been bored during task performance. Future studies should consider the use of gamified motor learning tasks to increase motivation (Alexandrovsky et al., 2019; Friehs et al., 2020). Our study design also assumed that anodal stimulation would result in cortical excitation, which may not be the case as described above. Finally, the size and placement of the electrodes used in the present study likely affected the spread of the current to other brain regions outside the targeted area (see ROAST output). However, the tDCS protocol used here was standard.

## Conclusion

Similar to what we reported with young adults (Greeley et al., 2020), we observed impaired sequence learning after the application of anodal tDCS over the left or right PFC for older adults. In combination, these two studies suggest a role for the bilateral prefrontal cortices in the early stages of sequence learning, regardless of age. Additionally, there was no instance where the application of anodal tDCS either over the right or left PFC, left M1, or SMC improved explicit motor sequence learning for older adults. These findings contribute to mounting evidence of the difficultly of using tDCS in an aging population and reveal a need for the field to adopt an individualized approach to non-invasive brain stimulation, especially in older adults. Recent work by Albizu et al. (2020) suggests that machine learning algorithms can use current density models to predict tDCS responsivity in older adults; such approaches will lead to better optimization of training interventions coupled with brain stimulation in the future.

## Data Availability Statement

The raw data supporting the conclusions of this article will be made available by the authors, without undue reservation.

## Ethics Statement

The studies involving human participants were reviewed and approved by IRB Ethics Board at the University of Michigan. The patients/participants provided their written informed consent to participate in this study.

## Author Contributions

BG was responsible for design, data collection, analysis, interpretation of results, and writing and preparation of the manuscript. JB was responsible for design, assistance with the chunking analysis and editing of the manuscript. WV and RS were jointly responsible for conceptualization (ideas, substance, and design) and manuscript edits. All authors contributed to the article and approved the submitted version.

## Conflict of Interest

The authors declare that the research was conducted in the absence of any commercial or financial relationships that could be construed as a potential conflict of interest.

## Publisher’s Note

All claims expressed in this article are solely those of the authors and do not necessarily represent those of their affiliated organizations, or those of the publisher, the editors and the reviewers. Any product that may be evaluated in this article, or claim that may be made by its manufacturer, is not guaranteed or endorsed by the publisher.

## References

[B1] Acuna D. E. Wymbs N. F. Reynolds C. A. Picard N. Turner R. S. Strick P. L. (2014). Multifaceted aspects of chunking enable robust algorithms. *J. Neurophysiol.* 112 1849–1856. 10.1152/jn.00028.2014 25080566 PMC4200007

[B2] Ahn H. Zhong C. Miao H. Chaoul A. Park L. Yen I. H. (2019). Efficacy of combining home-based transcranial direct current stimulation with mindfulness-based meditation for pain in older adults with knee osteoarthritis: a randomized controlled pilot study. *J. Clin. Neurosci.* 70 140–145. 10.1016/j.jocn.2019.08.047 31421990

[B3] Albizu A. Fang R. Indahlastari A. O’Shea A. Stolte S. E. See K. B. (2020). Machine learning and individual variability in electric field characteristics predict tDCS treatment response. *Brain Stimul.* 13 1753–1764. 10.1016/j.brs.2020.10.001 33049412 PMC7731513

[B4] Alexandrovsky D. Friehs M. A. Birk M. V. Yates R. K. Mandryk R. L. (2019). “Game dynamics that support snacking, not feasting,” in *Proceedings of the Annual Symposium on Computer-Human Interaction in Play*, Barcelona, 573–588.

[B5] Arnau J. Bono R. Blanca M. J. Bendayan R. (2012). Using the linear mixed model to analyze nonnormal data distributions in longitudinal designs. *Behav. Res. Methods* 44 1224–1238. 10.3758/s13428-012-0196-y 22399245

[B6] Beck A. T. Steer R. A. Carbin M. G. (1988). Psychometric properties of the beck depression inventory: twenty-five years of evaluation. *Clin. Psychol. Rev.* 8 77–100. 10.1016/0272-7358(88)90050-5

[B7] Bikson M. Datta A. Elwassif M. (2009). Establishing safety limits for transcranial direct current stimulation. *Clin. Neurophysiol.* 120:1033. 10.1016/j.clinph.2009.03.018 19394269 PMC2754807

[B8] Bo J. Seidler R. D. (2009). Visuospatial working memory capacity predicts the organization of acquired explicit motor sequences. *J. Neurophysiol.* 101 3116–3125. 10.1152/jn.00006.2009 19357338 PMC2694099

[B9] Bo J. Borza V. Seidler R. D. (2009). Age-related declines in visuospatial working memory correlate with deficits in explicit motor sequence learning. *J. Neurophysiol.* 102 2744–2754. 10.1152/jn.00393.2009 19726728 PMC2777814

[B10] Brown R. M. Robertson E. M. Press D. Z. (2009). Sequence skill acquisition and off-line learning in normal aging. *PLoS One* 4:e6683. 10.1371/journal.pone.0006683 19690610 PMC2723909

[B11] Cabeza R. (2002). Hemispheric asymmetry reduction in older adults: the HAROLD model. *Psychol. Aging* 17:85. 11931290 10.1037//0882-7974.17.1.85

[B12] Carson N. Leach L. Murphy K. J. (2018). A re-examination of Montreal cognitive assessment (MoCA) cutoff scores. *Int. J. Geriatr. Psychiatry* 33 379–388. 10.1002/gps.4756 28731508

[B13] Charvet L. E. Kasschau M. Datta A. Knotkova H. Stevens M. C. Alonzo A. (2015). Remotely-supervised transcranial direct current stimulation (tDCS) for clinical trials: guidelines for technology and protocols. *Front. Syst. Neurosci.* 9:26. 10.3389/fnsys.2015.00026 25852494 PMC4362220

[B14] Chow R. Noly-Gandon A. Moussard A. Ryan J. D. Alain C. (2021). Effects of transcranial direct current stimulation combined with listening to preferred music on memory in older adults. *Sci. Rep.* 11:12638. 10.1038/s41598-021-91977-8 34135392 PMC8209223

[B15] Doyon J. Benali H. (2005). Reorganization and plasticity in the adult brain during learning of motor skills. *Curr. Opin. Neurobiol.* 15 161–167. 10.1016/j.conb.2005.03.004 15831397

[B16] Dumel G. Bourassa M. E. Desjardins M. Voarino N. Charlebois-Plante C. Doyon J. (2016). Multisession anodal tDCS protocol improves motor system function in an aging population. *Neural Plast.* 2016:5961362. 10.1155/2016/5961362 26881118 PMC4736991

[B17] Ekstrom R. B. French J. W. Harman H. H. (1979). Cognitive factors: their identification and replication. *Multivariate Behav. Res. Monogr.* 79:84.

[B18] Esiri M. M. (2007). Ageing and the brain. *J. Pathol.* 211 181–187. 17200950 10.1002/path.2089

[B19] Farnad L. Ghasemian-Shirvan E. Mosayebi-Samani M. Kuo M. F. Nitsche M. A. (2021). Exploring and optimizing the neuroplastic effects of anodal transcranial direct current stimulation over the primary motor cortex of older humans. *Brain Stimul.* 14 622–634. 10.1016/j.brs.2021.03.013 33798763

[B20] Fertonani A. Brambilla M. Cotelli M. Miniussi C. (2014). The timing of cognitive plasticity in physiological aging: a tDCS study of naming. *Front. Aging Neurosci.* 6:131. 10.3389/fnagi.2014.00131 25009493 PMC4068214

[B21] Filmer H. L. Mattingley J. B. Dux P. E. (2020). Modulating brain activity and behaviour with tDCS: rumours of its death have been greatly exaggerated. *Cortex* 123 141–151. 10.1016/j.cortex.2019.10.006 31783223

[B22] Floyer-Lea A. Matthews P. M. (2005). Distinguishable brain activation networks for short-and long-term motor skill learning. *J. Neurophysiol.* 94 512–518. 10.1152/jn.00717.2004 15716371

[B23] Friehs M. A. Dechant M. Vedress S. Frings C. Mandryk R. L. (2020). Effective gamification of the stop-signal task: two controlled laboratory experiments. *JMIR Serious Games* 8:e17810. 10.2196/17810 32897233 PMC7509611

[B24] Fujiyama H. Hinder M. R. Barzideh A. Van de Vijver C. Badache A. C. Manrique-C M. N. (2017). Preconditioning tDCS facilitates subsequent tDCS effect on skill acquisition in older adults. *Neurobiol. Aging* 51 31–42. 28033506 10.1016/j.neurobiolaging.2016.11.012

[B25] Funahashi S. (2017). Working memory in the prefrontal cortex. *Brain Sci.* 7:49. 10.3390/brainsci7050049 PMC5447931 28448453

[B26] Galea J. M. Albert N. B. Ditye T. Miall R. C. (2010). Disruption of the dorsolateral prefrontal cortex facilitates the consolidation of procedural skills. *J. Cogn. Neurosci.* 22 1158–1164. 10.1162/jocn.2009.21259 19413472 PMC6010144

[B27] Greeley B. Seidler R. D. (2019). Differential effects of left and right prefrontal cortex anodal transcranial direct current stimulation during probabilistic sequence learning. *J. Neurophysiol.* 121 1906–1916. 10.1152/jn.00795.2018 30917064

[B28] Greeley B. Barnhoorn J. S. Verwey W. B. Seidler R. D. (2020). Multi-session transcranial direct current stimulation over primary motor cortex facilitates sequence learning, chunking, and one year retention. *Front. Hum. Neurosci.* 14:75. 10.3389/fnhum.2020.00075 32226370 PMC7080980

[B29] Habich A. Slotboom J. Peter J. Wiest R. Klöppel S. (2020). No effect of anodal tDCS on verbal episodic memory performance and neurotransmitter levels in young and elderly participants. *Neural Plast*. 2020:8896791. 10.1155/2020/8896791 33029128 PMC7528151

[B30] Hashemirad F. Zoghi M. Fitzgerald P. B. Jaberzadeh S. (2016). The effect of anodal transcranial direct current stimulation on motor sequence learning in healthy individuals: a systematic review and meta-analysis. *Brain Cogn.* 102 1–12. 10.1016/j.bandc.2015.11.005 26685088

[B31] Huang Y. Datta A. Bikson M. Parra L. C. (2019). Realistic volumetric-approach to simulate transcranial electric stimulation—ROAST—a fully automated open-source pipeline. *J. Neural Eng.* 16:056006. 10.1088/1741-2552/ab208d 31071686 PMC7328433

[B32] Huang Y. Liu A. A. Lafon B. Friedman D. Dayan M. Wang X. (2017). Measurements and models of electric fields in the in vivo human brain during transcranial electric stimulation. *elife* 6:e18834. 28169833 10.7554/eLife.18834 PMC5370189

[B33] Indahlastari A. Albizu A. O’Shea A. Forbes M. A. Nissim N. R. Kraft J. N. (2020). Modeling transcranial electrical stimulation in the aging brain. *Brain Stimul.* 13 664–674. 10.1016/j.brs.2020.02.007 32289695 PMC7196025

[B34] Iyer M. B. Mattu U. Grafman J. Lomarev M. Sato S. Wassermann E. M. (2005). Safety and cognitive effect of frontal DC brain polarization in healthy individuals. *Neurology* 64 872–875. 10.1212/01.WNL.0000152986.07469.E9 15753425

[B35] Janacsek K. Ambrus G. G. Paulus W. Antal A. Nemeth D. (2015). Right hemisphere advantage in statistical learning: evidence from a probabilistic sequence learning task. *Brain Stimul.* 8 277–282. 10.1016/j.brs.2014.11.008 25499036

[B36] Kane M. J. Engle R. W. (2002). The role of prefrontal cortex in working-memory capacity, executive attention, and general fluid intelligence: an individual-differences perspective. *Psychonom. Bull. Rev.* 9 637–671. 10.3758/bf03196323 12613671

[B37] Kasschau M. Reisner J. Sherman K. Bikson M. Datta A. Charvet L. E. (2016). Transcranial direct current stimulation is feasible for remotely supervised home delivery in multiple sclerosis. *Neuromodulation* 19 824–831. 10.1111/ner.12430 27089545

[B38] Kennerley S. W. Sakai K. Rushworth M. F. S. (2004). Organization of action sequences and the role of the pre-SMA. *J. Neurophysiol.* 91 978–993. 10.1152/jn.00651.2003 14573560

[B39] Kimura T. Kaneko F. Nagamine T. (2021). The effects of transcranial direct current stimulation on dual-task interference depend on the dual-task content. *Front. Hum. Neurosci.* 15:653713. 10.3389/fnhum.2021.653713 33841121 PMC8032873

[B40] Lee J. Y. Lee D. W. Cho S. J. Na D. L. Jeon H. J. Kim S. K. (2008). Brief screening for mild cognitive impairment in elderly outpatient clinic: validation of the Korean version of the Montreal Cognitive Assessment. *J. Geriatr. Psychiatry Neurol.* 21 104–110. 10.1177/0891988708316855 18474719

[B41] Lin C. H. J. Chiang M. C. Wu A. D. Iacoboni M. Udompholkul P. Yazdanshenas O. (2012). Age related differences in the neural substrates of motor sequence learning after interleaved and repetitive practice. *Neuroimage* 62 2007–2020. 10.1016/j.neuroimage.2012.05.015 22584226

[B42] Lo S. Andrews S. (2015). To transform or not to transform: using generalized linear mixed models to analyse reaction time data. *Front. Psychol.* 6:1171. 10.3389/fpsyg.2015.01171 26300841 PMC4528092

[B43] Luck S. J. Vogel E. K. (1997). The capacity of visual working memory for features and conjunctions. *Nature* 390 279–281. 9384378 10.1038/36846

[B44] Michely J. Volz L. J. Hoffstaedter F. Tittgemeyer M. Eickhoff S. B. Fink G. R. (2018). Network connectivity of motor control in the ageing brain. *NeuroImage Clin.* 18 443–455. 10.1016/j.nicl.2018.02.001 29552486 PMC5852391

[B45] Monte-Silva K. Kuo M. F. Hessenthaler S. Fresnoza S. Liebetanz D. Paulus W. (2013). Induction of late LTP-like plasticity in the human motor cortex by repeated non-invasive brain stimulation. *Brain Stimul.* 6 424–432. 10.1016/j.brs.2012.04.011 22695026

[B46] Mooney R. A. Cirillo J. Byblow W. D. (2019). Neurophysiological mechanisms underlying motor skill learning in young and older adults. *Exp. Brain Res.* 237 2331–2344. 10.1007/s00221-019-05599-8 31289887

[B47] Muffel T. Kirsch F. Shih P. C. Kalloch B. Schaumberg S. Villringer A. (2019). Anodal transcranial direct current stimulation over S1 differentially modulates proprioceptive accuracy in young and old adults. *Front. Aging Neurosci.* 11:264. 10.3389/fnagi.2019.00264 31611782 PMC6775783

[B48] Nasreddine Z. S. Phillips N. A. Bédirian V. Charbonneau S. Whitehead V. Collin I. (2005). The montreal cognitive assessment, MoCA: a brief screening tool for mild cognitive impairment. *J. Am. Geriatr Soc.* 53, 695–699. 10.1111/j.1532-5415.2005.53221.x 15817019

[B49] Nissim N. R. O’Shea A. Indahlastari A. Telles R. Richards L. Porges E. (2019). Effects of in-scanner bilateral frontal tDCS on functional connectivity of the working memory network in older adults. *Front. Aging Neurosci.* 11:51. 10.3389/fnagi.2019.00051 30930766 PMC6428720

[B50] Nitsche M. A. Doemkes S. Karakose T. Antal A. Liebetanz D. Lang N. (2007). Shaping the effects of transcranial direct current stimulation of the human motor cortex. *J. Neurophysiol.* 97 3109–3117. 10.1152/jn.01312.2006 17251360

[B51] Nomura T. Kirimoto H. (2018). Anodal transcranial direct current stimulation over the supplementary motor area improves anticipatory postural adjustments in older adults. *Front. Hum. Neurosci.* 12:317. 10.3389/fnhum.2018.00317 30123118 PMC6086140

[B52] Oldfield R. C. (1971). The assessment and analysis of handedness: the Edinburgh inventory. *Neuropsychologia* 9 97–113. 10.1016/0028-3932(71)90067-4 5146491

[B53] Pammi V. C. Miyapuram K. P. Samejima K. Bapi R. S. Doya K. (2012). Changing the structure of complex visuo-motor sequences selectively activates the fronto-parietal network. *Neuroimage* 59 1180–1189. 10.1016/j.neuroimage.2011.08.006 21867758

[B54] Patel R. Ashcroft J. Patel A. Ashrafian H. Woods A. J. Singh H. (2019). The impact of transcranial direct current stimulation on upper-limb motor performance in healthy adults: a systematic review and meta-analysis. *Front. Neurosci.* 13:1213. 10.3389/fnins.2019.01213 31803003 PMC6873898

[B55] Przybyla A. Haaland K. Y. Bagesteiro L. B. Sainburg R. L. (2011). Motor asymmetry reduction in older adults. *Neurosci. Lett.* 489 99–104. 21144883 10.1016/j.neulet.2010.11.074 PMC3422634

[B56] Raz N. (2000). “Aging of the brain and its impact on cognitive performance: integration of structural and functional findings,” in *The Handbook of Aging and Cognition*, eds Craik F. I. M. Salthouse T. A. (Mahwah, NJ: Lawrence Erlbaum Associates Publishers), 1–90.

[B57] Reis J. Schambra H. M. Cohen L. G. Buch E. R. Fritsch B. Zarahn E. (2009). Noninvasive cortical stimulation enhances motor skill acquisition over multiple days through an effect on consolidation. *Proc. Natl. Acad. Sci. U.S.A.* 106 1590–1595. 10.1073/pnas.0805413106 19164589 PMC2635787

[B58] Rossi S. Miniussi C. Pasqualetti P. Babiloni C. Rossini P. M. Cappa S. F. (2004). Age-related functional changes of prefrontal cortex in long-term memory: a repetitive transcranial magnetic stimulation study. *J. Neurosci.* 24 7939–7944. 10.1523/JNEUROSCI.0703-04.2004 15356207 PMC6729939

[B59] Ruitenberg M. F. Verwey W. B. Schutter D. J. Abrahamse E. L. (2014). Cognitive and neural foundations of discrete sequence skill: a TMS study. *Neuropsychologia* 56 229–238. 10.1016/j.neuropsychologia.2014.01.014 24486768

[B60] Rumpf J. J. Wegscheider M. Hinselmann K. Fricke C. King B. R. Weise D. (2017). Enhancement of motor consolidation by post-training transcranial direct current stimulation in older people. *Neurobiol. Aging* 49 1–8. 10.1016/j.neurobiolaging.2016.09.003 27723499

[B61] Saucedo Marquez C. M. Zhang X. Swinnen S. P. Meesen R. Wenderoth N. (2013). Task-specific effect of transcranial direct current stimulation on motor learning. *Front. Hum. Neurosci.* 7:333. 10.3389/fnhum.2013.00333 23847505 PMC3696911

[B62] Scahill R. I. Frost C. Jenkins R. Whitwell J. L. Rossor M. N. Fox N. C. (2003). A longitudinal study of brain volume changes in normal aging using serial registered magnetic resonance imaging. *Arch. Neurol.* 60 989–994. 10.1001/archneur.60.7.989 12873856

[B63] Schielzeth H. Dingemanse N. J. Nakagawa S. Westneat D. F. Allegue H. Teplitsky C. (2020). Robustness of linear mixed-effects models to violations of distributional assumptions. *Methods Ecol. Evol.* 11 1141–1152.

[B64] Seidler R. D. (2006). Differential effects of age on sequence learning and sensorimotor adaptation. *Brain Res. Bull.* 70 337–346. 10.1016/j.brainresbull.2006.06.008 17027769

[B65] Seidler R. D. Bernard J. A. Burutolu T. B. Fling B. W. Gordon M. T. Gwin J. T. (2010). Motor control and aging: links to age-related brain structural, functional, and biochemical effects. *Neurosci. Biobehav. Rev.* 34 721–733. 10.1016/j.neubiorev.2009.10.005 19850077 PMC2838968

[B66] Seidler R. D. Gluskin B. S. Greeley B. (2017). Right prefrontal cortex transcranial direct current stimulation enhances multi-day savings in sensorimotor adaptation. *J. Neurophysiol.* 117 429–435. 10.1152/jn.00563.2016 27832598 PMC5253398

[B67] Shekhawat G. S. Vanneste S. (2018). Optimization of transcranial direct current stimulation of dorsolateral prefrontal cortex for tinnitus: a non-linear dose-response effect. *Sci. Rep.* 8:8311. 10.1038/s41598-018-26665-1 29844532 PMC5974180

[B68] Song S. Zilverstand A. Gui W. Li H. J. Zhou X. (2019). Effects of single-session versus multi-session non-invasive brain stimulation on craving and consumption in individuals with drug addiction, eating disorders or obesity: a meta-analysis. *Brain Stimul.* 12 606–618. 10.1016/j.brs.2018.12.975 30612944

[B69] Stagg C. J. Jayaram G. Pastor D. Kincses Z. T. Matthews P. M. Johansen-Berg H. (2011). Polarity and timing-dependent effects of transcranial direct current stimulation in explicit motor learning. *Neuropsychologia* 49 800–804. 10.1016/j.neuropsychologia.2011.02.009 21335013 PMC3083512

[B70] Talsma L. J. Kroese H. A. Slagter H. A. (2017). Boosting cognition: effects of multiple-session transcranial direct current stimulation on working memory. *J. Cogn. Neurosci.* 29 755–768. 10.1162/jocn_a_01077 27897670

[B71] Tiffin J. Asher E. J. (1948). The Purdue pegboard: norms and studies of reliability and validity. *J. Appl. Psychol.* 32:234. 10.1037/h0061266 18867059

[B72] Verwey W. B. (2010). Diminished motor skill development in elderly: indications for limited motor chunk use. *Acta Psychol.* 134 206–214. 10.1016/j.actpsy.2010.02.001 20189547

[B73] Verwey W. B. Eikelboom T. (2003). Evidence for lasting sequence segmentation in the discrete sequence-production task. *J. Motor Behav.* 35 171–181. 10.1080/00222890309602131 12711587

[B74] Verwey W. B. Jouen A. L. Dominey P. F. Ventre-Dominey J. (2019). Explaining the neural activity distribution associated with discrete movement sequences: evidence for parallel functional systems. *Cogn. Affect. Behav. Neurosci.* 19 138–153. 10.3758/s13415-018-00651-6 30406305 PMC6344389

[B75] Vollmann H. Conde V. Sewerin S. Taubert M. Sehm B. Witte O. W. (2013). Anodal transcranial direct current stimulation (tDCS) over supplementary motor area (SMA) but not pre-SMA promotes short-term visuomotor learning. *Brain Stimul.* 6 101–107. 10.1016/j.brs.2012.03.018 22659022

[B76] Waters S. Wiestler T. Diedrichsen J. (2017). Cooperation not competition: bihemispheric tDCS and fMRI show role for ipsilateral hemisphere in motor learning. *J. Neurosci.* 37 7500–7512. 28674174 10.1523/JNEUROSCI.3414-16.2017 PMC5546115

[B77] Waters-Metenier S. Husain M. Wiestler T. Diedrichsen J. (2014). Bihemispheric transcranial direct current stimulation enhances effector-independent representations of motor synergy and sequence learning. *J. Neurosci.* 34 1037–1050. 10.1523/JNEUROSCI.2282-13.2014 24431461 PMC3891947

[B78] Wechsler D. (1958). *The Measurement and Appraisal of Adult Intelligence*, 4th Edn. Philadelphia, PA: Williams & Wilkins Co.

[B79] Wilhelm I. Diekelmann S. Born J. (2008). Sleep in children improves memory performance on declarative but not procedural tasks. *Learn. Mem.* 15 373–377. 10.1101/lm.803708 18441295

[B80] Wilhelm I. Prehn-Kristensen A. Born J. (2012). Sleep-dependent memory consolidation–what can be learnt from children? *Neurosci. Biobehav. Rev.* 36 1718–1728. 10.1016/j.neubiorev.2012.03.002 22430027

[B81] Wu T. Hallett M. (2005). The influence of normal human ageing on automatic movements. *J. Physiol.* 562 605–615. 10.1113/jphysiol.2004.076042 15513939 PMC1665504

[B82] Wymbs N. F. Bassett D. S. Mucha P. J. Porter M. A. Grafton S. T. (2012). Differential recruitment of the sensorimotor putamen and frontoparietal cortex during motor chunking in humans. *Neuron* 74 936–946. 10.1016/j.neuron.2012.03.038 22681696 PMC3372854

[B83] Zhu F. F. Yeung A. Y. Poolton J. M. Lee T. M. Leung G. K. Masters R. S. (2015). Cathodal transcranial direct current stimulation over left dorsolateral prefrontal cortex area promotes implicit motor learning in a golf putting task. *Brain Stimul.* 8 784–786. 10.1016/j.brs.2015.02.005 25857398

[B84] Zimerman M. Heise K. F. Gerloff C. Cohen L. G. Hummel F. C. (2014). Disrupting the ipsilateral motor cortex interferes with training of a complex motor task in older adults. *Cereb. Cortex* 24 1030–1036. 10.1093/cercor/bhs385 23242199

[B85] Zimerman M. Nitsch M. Giraux P. Gerloff C. Cohen L. G. Hummel F. C. (2013). Neuroenhancement of the aging brain: restoring skill acquisition in old subjects. *Ann. Neurol.* 73:10. 10.1002/ana.23761 23225625 PMC4880032

