# Supplementary material for: Anodal Transcranial Direct Current Stimulation Over Prefrontal Cortex Slows Sequence Learning in Older Adults
Source: Front Hum Neurosci. 2022 Feb 24;16:814204. doi: 10.3389/fnhum.2022.814204 (PMC8907426; doi:10.3389/fnhum.2022.814204)
Supplement: Supplementary file 1 [file Data_Sheet_1.PDF]

Methods

Side effects questionnaire

At the end of sessions one and two, participants were given a custom made tDCS side effects questionnaire. We asked participants whether they experienced any of the following symptoms or side effects: headache, neck pain, scalp pain, scalp burns, tingling, skin redness, sleepiness, trouble concentrating, and acute mood change, and other (specify). Participants could then indicate the severity of each symptom or side effect by indicating whether each one was absent, mild, moderate, or severe. We coded absent as 0, mild as 1, moderate as 2, and severe as 3.

Data analysis

We ran a series of Mann-Whitney U tests comparing each active tDCS condition (right PFC, left PFC, left M1, and SMC) to sham for each symptom / side effect.

Results

We had side effect results from the following tDCS groups: M1 (n = 10), right PFC (n = 9), left PFC (n = 9), SMC (n = 10), and sham (n = 8).

Session 1

There was a significant difference between the sham and left M1 older adult tDCS groups for the reported side effect of sleepiness ($p = .005$). Specifically, participants in the left M1 group reported absent sleepiness (M = 0.00, SD = .00) whereas the sham group reported having experienced mild sleepiness (M = 1.00, SD = .93). All other comparisons did not reach statistical significance in session 1 ($p > .06$). See Supplemental Table 1 for averages for all tDCS groups.

Session 2

There was a significant difference between the sham and the SMC older adult tDCS groups for the reported side effect trouble concentrating ($p = .021$). Specifically, participants in the SMC group reported an absence of having trouble concentrating (M = 0.10, SD = .32) whereas the sham group reported having somewhere between absent and mild trouble concentrating (M = 0.75, SD = .71). All other comparisons did not reach significance in session 1 ($p > .07$). See Supplemental Table 1 for averages for all tDCS groups.

Supplemental Table 1. Averaged reported side effects or symptoms from sessions 1 and 2 for all older adult tDCS groups. Immediately following each tDCS session, participants received a custom side effects questionnaire regarding symptoms they may have experienced. Participants indicated their symptom severity by checking columns to indicate absent, mild, moderate, or severe. We coded absent as 0, mild as 1, moderate as 2, and severe as 3. Values are reported with mean and standard deviation in parentheses.

**Session**	**Symptom**	**tDCS Group**				
		**Right PFC**	**Left PFC**	**Left M1**	**SMC**	**Sham**
**Session 1**	**Headache**	0.00 (.00)	0.00 (.35)	0.10(.32)	0.10 (.35)	0.13 (.35)
	**Neck Pain**	0.00 (.00)	0.00 (.35)	0.00 (.00)	0.00 (.35)	0.13 (.35)
	**Scalp Pain**	0.11 (.33)	0.11 (.74)	0.10 (.32)	0.20 (.74)	0.38 (.74)
	**Scalp Burns**	0.00 (.00)	0.11 (.35)	0.10 (.32)	0.30 (.35)	0.13 (.35)
	**Tingling**	0.78 (.44)	0.89 (.83)	0.60 (.70)	0.50 (.83)	1.13 (.83)
	**Skin Redness**	0.00 (.00)	0.22 (.35)	0.30 (.67)	0.00 (.35)	0.13 (.35)
	**Sleepiness**	0.44 (.73)	0.22 (.93)	0.00 (.00)	0.30 (.93)	1.00 (.93)
	**Trouble Concentrating**	0.11 (.33)	0.44 (.76)	0.10 (.32)	0.20 (.76)	0.50 (.76)
	**Acute Mood Change**	0.00 (.00)	0.11 (.71)	0.10 (.32)	0.00 (.71)	0.25 (.71)
**Session 2**	**Headache**	0.00 (.00)	0.00 (.00)	0.00 (.00)	0.40 (.52)	0.38 (.74)
	**Neck Pain**	0.00 (.00)	0.00 (.00)	0.00 (.00)	0.00 (.00)	0.00 (.00)
	**Scalp Pain**	0.00 (.00)	0.00 (.00)	0.36 (.92)	0.00 (.00)	0.25 (.46)
	**Scalp Burns**	0.00 (.00)	0.00 (.00)	0.18 (.60)	0.20 (.42)	0.13 (.35)
	**Tingling**	0.56 (.53)	0.89 (.60)	0.73 (.65)	0.40 (.52)	0.75 (.71)
	**Skin Redness**	0.00 (.00)	0.00 (.00)	0.00 (.00)	0.00 (.00)	0.13 (.35)
	**Sleepiness**	0.11 (.33)	0.22 (.44)	0.18 (.40)	0.10 (.32)	0.75 (.89)
	**Trouble Concentrating**	0.22 (.44)	0.33 (.50)	0.27 (.47)	0.10 (.32)	0.75 (.71)
	**Acute Mood Change**	0.00 (.00)	0.00 (.00)	0.27 (.65)	0.00 (.00)	0.13 (.35)
